# Microbial Polysaccharides Extracted from Different Mature Muds of the Euganean Thermal District Show Similar Anti-Inflammatory Activity In Vivo

**DOI:** 10.3390/ijms25094999

**Published:** 2024-05-03

**Authors:** Micol Caichiolo, Raffaella Margherita Zampieri, Alessandra Adessi, Matilde Ciani, Fabrizio Caldara, Luisa Dalla Valle, Nicoletta La Rocca

**Affiliations:** 1 Department of Biology, University of Padova, Via U. Bassi 58/b, 35131 Padova, Italy; micol.caichiolo@phd.unipd.it (M.C.); raffaellamargherita.zampieri@phd.unipd.it (R.M.Z.); nicoletta.larocca@unipd.it (N.L.R.); 2 Research Institute on Terrestrial Ecosystems (IRET), National Research Council (CNR), Via Madonna del Piano 10, 50019 Firenze, Italy; 3 Department of Agriculture, Food, Environment and Forest (DAGRI), University of Florence, Via Maragliano 77, 50144 Firenze, Italy; alessandra.adessi@unifi.it (A.A.); matilde.ciani@unifi.it (M.C.); 4 Pietro D’Abano Thermal Studies Center, Via Jappelli 5, 35031 Padova, Italy; fabrizio.caldara@centrostuditermali.org

**Keywords:** thermal mud, peloid, microbiota, cyanobacteria, inflammation, zebrafish, polysaccharide, bioactive molecule

## Abstract

The Euganean Thermal District, situated in North-East Italy, is one of Europe’s largest and oldest thermal centres. The topical application of its therapeutic thermal muds is recognised by the Italian Health System as a beneficial treatment for patients suffering from arthro-rheumatic diseases. Polysaccharides produced by the mud microbiota have been recently identified as anti-inflammatory bioactive molecules. In this paper we analysed the efficacy of Microbial-Polysaccharides (M-PS) derived from mature muds obtained at different maturation temperatures, both within and outside the codified traditional mud maturation range. M-PSs were extracted from six mature muds produced by five spas of the Euganean Thermal District and investigated for their chemical properties, monosaccharide composition and in vivo anti-inflammatory potential, using the zebrafish model organism. Additionally, mature muds were characterized for their microbiota composition using Next-Generation Sequencing. The results showed that all M-PSs exhibit similar anti-inflammatory potential, referable to their comparable chemical composition. This consistency was observed despite changes in cyanobacteria populations, suggesting a possible role of the entire microbial community in shaping the properties of these biomolecules. These findings highlight the importance of scientific research in untangling the origins of the therapeutic efficacy of Euganean Thermal muds in the treatment of chronic inflammatory conditions.

## 1. Introduction

The Euganean Thermal District, situated in Padova, Italy, is one of the largest and oldest thermal centres in Europe. The topical application of its therapeutic thermal muds, also called pelotherapy, is recognised by the Italian Health System as a healing treatment for patients suffering from arthro-rheumatic diseases. This beneficial mud is obtained following a specific protocol, codified by regional rules [1], that allows the thermal structures, or spas, to obtain the “Mature Mud AOC” certification. Specifically, the virgin clay is collected from thermal lakes in the Euganean Area and distributed among spas where it undergoes maturation, in concrete ponds or steel tanks, covered by flowing thermal waters at nearly 40 °C for a period of at least two months. During this time, a thick green biofilm, consisting of microorganisms embedded in a matrix composed of extracellular polymeric substances (EPSs), is formed, indicating a proper mud maturation. At this point, the mature mud is mixed and transferred to ponds with thermal water at nearly 60 °C (6–24 h) to diminish the natural microbial load and help to fluidify the product before its use for human applications (therapeutic mud).

As mud matures, therapeutically active compounds may form through metabolic synthesis and degradation of organic molecules carried out by various microorganisms. These molecules have the capacity to enhance the healing effects of the peloid, known for its stimulating, anti-inflammatory, and analgesic properties [2]. Indeed, it is known that the therapeutic efficacy of peloids, and in our case of the Euganean muds, is not solely attributed to heat or electrolytes of the thermal mineral water but also to bioactive molecules produced by microorganisms that colonize the mud surface [3]. Specifically, lipids and polysaccharides produced by *Phormidium* sp. ETS-05, the most abundant Cyanobacteria species present in the mud maturated following traditional conditions, have already been investigated for their antioxidant and anti-inflammatory capabilities [4,5,6,7]. For this reason, *Phormidium* sp. ETS-05 is considered the target species of Euganean muds.

However, analysis of mud maturation parameters and microbiota composition across 23 spas of the Euganean District, revealed differences in water temperature among thermal structures and even within ponds of the same spa, influencing the final microbial communities. The variability of this parameter can be attributed to the challenge faced by spa operators in regulating and maintaining a constant temperature in ponds. Indeed, in the traditional maturation system, thermal water from the hot spring at 75 °C is cooled in a settling pond before being distributed to ponds, typically connected in series. This process creates temperature variations, leading to deviations from the desired conditions (Figure S1). Nevertheless, a stable microbial community, predominantly composed of Cyanobacteria, Chloroflexi, Bacteroidetes, and Proteobacteria in various proportions, is established at a maturation temperature ranging between 37 and 47 °C. Water temperature has been identified as the primary environmental factor driving changes in Cyanobacteria abundance. In particular, it has been shown that a high presence of cyanobacteria in the microbiota, with *Phormidium sp.* ETS-05 as the main species, is distinctive of a mud maturated according to the traditional rules, whereas a complete shift in the cyanobacterial composition is observed at higher temperatures, characterized by a consistent increase in the abundance of the species *Thermospirulina andreolii* ETS-09 and *Leptolyngbyaceae* sp. ETS-13 [8,9]. This suggests that a study of the effects of the microbial composition changes on therapeutic mud properties is of high importance.

Cyanobacteria are the most abundant extremophilic organisms, composing microbial biofilms, as also found in other hot spring microbial mats around the world [10]. They are known to produce a large variety of molecules that exhibit beneficial properties with high potential in various fields, thanks to their antimicrobial, anti-inflammatory, and antioxidant activities [11,12].

Among these bioactive compounds, polysaccharides, complex heteropolymers characterized by a high molecular weight and varying composition, typically consisting of 3 to 10 monosaccharides, have been the focus of numerous studies investigating their biological activities. These studies included in vitro and in vivo testing of their beneficial properties [13]. Moreover, anti-inflammatory capabilities were demonstrated for hydrophilic mud extracts from Greece and Israel spa resorts [14] and cyanobacterial extracts derived from France Thermal Mud of Balaruc-Les-Bains [15].

In 2022, the anti-inflammatory potential of polysaccharides extracted directly from Euganean therapeutic mud was tested in vivo for the first time, using the model organism zebrafish. These polysaccharides were extracted from a therapeutic mud produced following the traditional maturation process at approximately 40 °C. Their efficacy was demonstrated through different zebrafish inflammation models and various experiments were conducted, including morphometric analyses of zebrafish developmental traits, evaluation of immune cell recruitment, and analyses of inflammatory marker gene expression via RT-qPCR. Overall, the study’s findings underscored the importance of polysaccharide components for the therapeutic efficacy of mud treatments [16].

In the complex context of the production of mature muds in the thermal sites of the Euganean Thermal District, muds matured at different conditions have never been tested for the anti-inflammatory capabilities of their bioactive compounds. Thus, in this work polysaccharides (Microbial-Polysaccharides, hereafter M-PS) were extracted from six mature muds produced by five spas of the Euganean Thermal District and were investigated for their chemical properties, monosaccharide composition, and beneficial activities in vivo, using the zebrafish, a model organism increasingly used for in vivo drug screening [17,18,19]. Additionally, microbial biodiversity of sampled muds was assessed through Next-Generation Sequencing (NGS) methods, with a focus on cyanobacterial species.

The purpose of this study was to determine the presence of possible differences in therapeutic efficacy among M-PSs derived from muds obtained at different maturation temperatures, highlighting the importance of scientific research in untangling the origins of the therapeutic efficacy of Euganean Thermal muds in the treatment of chronic inflammatory conditions.

## 2. Results and Discussion

### 2.1. Mud Maturation Collection Sites

In this study, mature mud samples were gathered from concrete ponds in five different spas within the Euganean Thermal District. Specifically, we chose three mature mud samples obtained from a temperature range of 37 to 47 °C, where *Phormidium* sp. ETS-05 is anticipated to be the most abundant species, and three mature mud samples obtained at higher temperatures, ranging from 49 to 54 °C. At higher temperatures species like *Thermospirulina andreolii* ETS-09 and *Leptolyngbyaceae* sp. ETS-13 are expected to be more abundant.

For each sampling site, Table 1 reports temperature of maturation, pH and conductivity of the water, and the presence of protective roofs. Muds from P-3 (Pond-3) and P-6 were sampled from different ponds of the same spa. In Figure S2 are reported pictures of the thermal environment of the sampled ponds.

Chlorophyll *a* and carotenoid contents in the mud samples are in agreement with the values obtained by Gris et al. [9] and Calderan et al. [20] (Table 1). Indeed, in Gris et al., the mean value of Chlorophyll content, measured considering all sampled sites, was 20.9 µg/g_MUD_ [9].

### 2.2. Mature Mud Microbial Community Composition

The abundance of cyanobacteria species was assessed using light and fluorescence microscopic techniques. Findings highlighted the variation in population composition among samples, with *Phormidium* sp. ETS-05 predominantly present in the medium range of temperature for this environment. At temperatures higher than 49 °C, there was a complete shift in cyanobacteria species, accompanied by a reduction in biodiversity, as illustrated in Figure S3.

To confirm and better characterize the microbiota composition, we performed high-throughput sequencing of the V4 region of the 16S rRNA gene. The number of raw reads obtained per sample ranged between ~240,000 and ~500,000. According to the assessment of species richness (alpha diversity), the coverage was enough to reach the saturation level and to obtain a complete overview of the taxa present (Figure S4). The 16S rRNA sequences of Amplicon Sequence Variants (ASVs) corresponding to the most abundant taxa and Cyanobacteria (abundance higher than 0.01%), are reported in Table S1.

**Figure 1:**
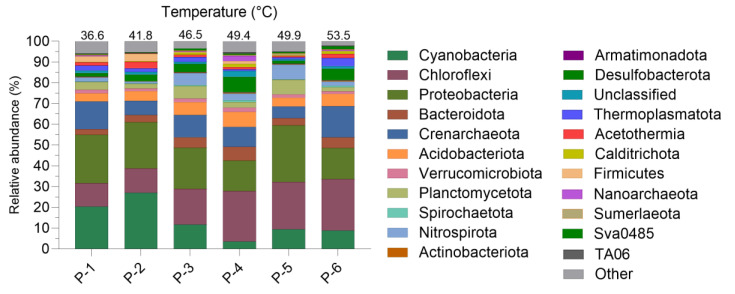
Relative abundance of the main phyla identified in the mature mud microbiota at different temperatures and sampling sites. Phyla with abundance lower than 0.05% were grouped and are displayed as Other.

The overall community composition remains largely consistent among mature mud samples, characterized by the presence of six main phyla (Cyanobacteria, Chloroflexi, Proteobacteria, Bacteroidota, Crenarchaeota and Acidobacteriota), which collectively account for approximately 66% to 76% of the total community (Figure 1). These results confirm the data reported by Gris et al. [9], indicating a consistency in the biodiversity found in this specific environment throughout the years.

**Figure 2:**
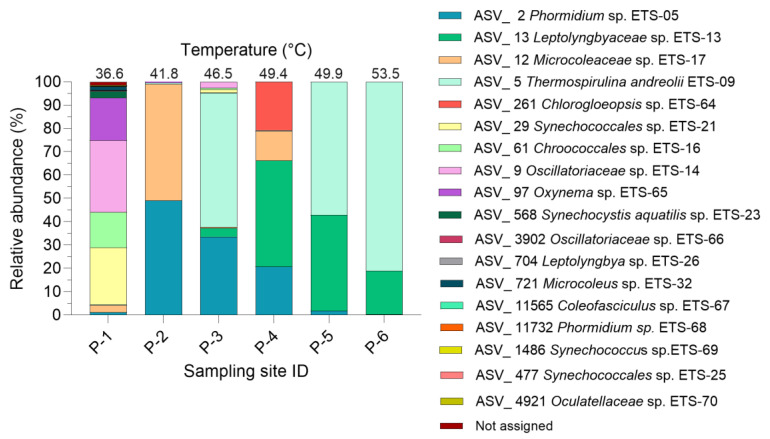
Relative abundance of the main Cyanobacteria ASVs identified in the mature mud microbiota at different temperatures and sampling sites. Only Cyanobacteria with abundance higher than 0.01% are shown.

The NGS detailed analysis of the Cyanobacteria phylum (3.5—27.1% of the microbiota) revealed the presence of 20 main ASVs (Figure 2). Eleven of the ASVs were assigned to species, and related ETS (Euganean Thermal Springs) code, already identified by Gris et al. [9]. Two unassigned sequences were matched to species isolated from thermal environments and hot springs (BLAST identity > 95%) and denoted with the genus name and a new ETS code (ETS-64; ETS-69). In case of uncertainty the family name followed by a new ETS code was used (ETS-66; ETS-70).

*Phormidium* sp. ETS-05 was found to be the most abundant species (abundance higher than 20%) in samples P-2, P-3 and P-4, corresponding to maturation temperatures from 41.8 to 49.4 °C. It’s worth noting that its presence diminishes as the temperature increases, dropping from around 50% in sample P-2 to 0.2% in sample P-6. The prevalence of *Phormidium* sp. ETS-05 also correlates with its optimal growth temperature (between 40–45 °C), in laboratory conditions, as recently determined by Zampieri et al. [21].

Meanwhile, species like *Leptolyngbyaceae* sp. ETS-13 and *Thermospirulina andreolii* ETS-09 become more abundant, comprising approximately 45 to 100% of the cyanobacteria population in samples at temperatures higher than 46 °C, producing a drastic drop in biodiversity. Moreover, cyanobacteria biodiversity increases at the lower temperature, as expected [9].

NGS analyses once again, as already highlighted by Gris et al. [9], revealed a relatively stable microbial community at the phylum level. However, there were significant fluctuations in the abundance of Cyanobacteria strains in response to temperature variations. Interestingly, other studies on thermal environments and hot springs microbial mats have also demonstrated the pivotal role of temperature in shaping the composition of microbial communities, particularly in regard to the relative abundance of Cyanobacteria [22,23,24].

### 2.3. Microbial Polysaccharides (M-PS) Characterization

In this study, to highlight potential differences in polysaccharide characteristics and bioactivity, we chose to sample the upper part of mature muds produced at different maturation temperatures. In this layer, microorganisms and cyanobacteria, whose strain abundance varies with temperature, are particularly abundant and release exopolysaccharide moieties. In particular, the previously studied therapeutic mud was obtained by mixing the entire amount of mud in a 39 °C pond and heating it at 60 °C for 6–24 h [16]. Instead, in this study, by sampling the top layer of mature muds, we focused on the area most affected by the microbial diversity and where bioactive molecules are produced.

M-PS were extracted from fresh matured mud samples using Na_2_EDTA and their polysaccharide contents were subsequently quantified using the Dubois method (Table S2). These molecules were then subjected to characterization of their composition and evaluation of their anti-inflammatory activity in vivo.

#### 2.3.1. FT-IR Spectra of M-PS

**Figure 3:**
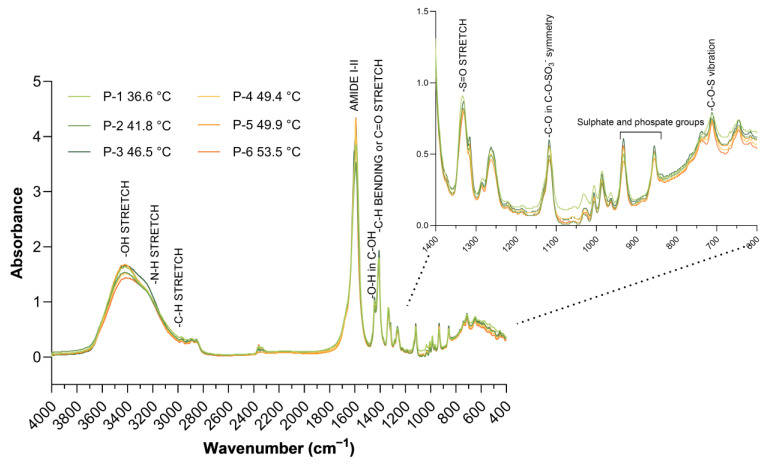
The FT-IR spectra of M-PSs extracted from mature muds, pressed into KBr pellets. With different colours are indicated M-PSs of different spas with the relative temperature of maturation of their muds.

FT-IR spectra of the six tested M-PSs overlap almost perfectly, indicating a compositional homogeneity of the extracted molecules (Figure 3). In particular, in the region 4000–2500 cm^−1^, the spectra showed a strong and wide band around 3400 cm^−1^ due to O-H stretching, a shoulder at 3250 cm^−1^ possibly related to N-H stretching and a moderate band around 2900 cm^−1^ assigned to asymmetric stretching vibrations of skeletal CH and CH_2_ [25]. The peak around 1600 cm^−1^ could be due to stretching vibration of C=O and C–N (Amide I) or to stretching vibration of C–N and deformation vibration of N–H (Amide II) [26]. The presence of amide groups confirms that the polymer is not only composed of polysaccharides, but also comprises some peptides and/or proteins associated with the M-PS [27]. The two smaller bands around 1400 cm^−1^ can be attributed to deformations in C-OH groups [25] and to the C-H bending of aliphatic CH_2_, even though the stretching of C=O from carboxylates may also contribute to the spectral features in this region [26,28]. Bands around 1300 cm^−1^highlight an asymmetric S=O stretching vibration whereas absorption around 1100 cm^−1^ refers to a symmetric C–O vibration which is specific to C–O–SO_3_ group, indicating characteristic absorptions of sulphate groups [27]. Moreover, even if the region with bands comprised between 930 and 840 cm^−1^ is of hard interpretation due to possible peaks overlapping, the stretches are likely due to α glycosidic bond and C-O-S vibration [27,29]. Finally, the band at 710 cm^−1^ could be attributed to asymmetrical and symmetrical C-O-S vibration [30].

Interestingly, FT-IR spectra are also similar to the one obtained by Zampieri et al. [16], in which polysaccharides were extracted from a therapeutic mud maturated at 39 °C. This indicates and confirms a specific and unique fingerprint of molecules extracted from muds of the Euganean Thermal District, despite the presence of changes in microbial community composition, especially in cyanobacteria species, caused by temperature variations.

#### 2.3.2. Zeta Potential of M-PS

The zeta potential, an index of intensity of electrostatic (charge) attraction between particles, showed that mature mud M-PSs were anionic. The zeta potential of each tested sample is listed in Table 2. On average it is attested to −22 ± 1.52 mV. The negative charge may be due to the presence of anionic groups (e.g., COO-, C-O-, SO_4_^2-^) and to the presence of glucuronic and galacturonic acid detected by the FT-IR and monosaccharide’s analyses. The results obtained agree with several studies that showed that polysaccharides with a net negative charge ameliorates their water solubility and activity as an ion exchange matrix of cations. In particular, polysaccharides that contains the functional groups and the characteristics mentioned above are able to reduce redox potential, stabilise the oxidation form of metal ions, prevent the production of excessive free radicals and protect the body from oxidative stress by chelating metal ions [31,32].

#### 2.3.3. M-PS’s Monosaccharide and Sulphate Groups Composition

**Figure 4:**
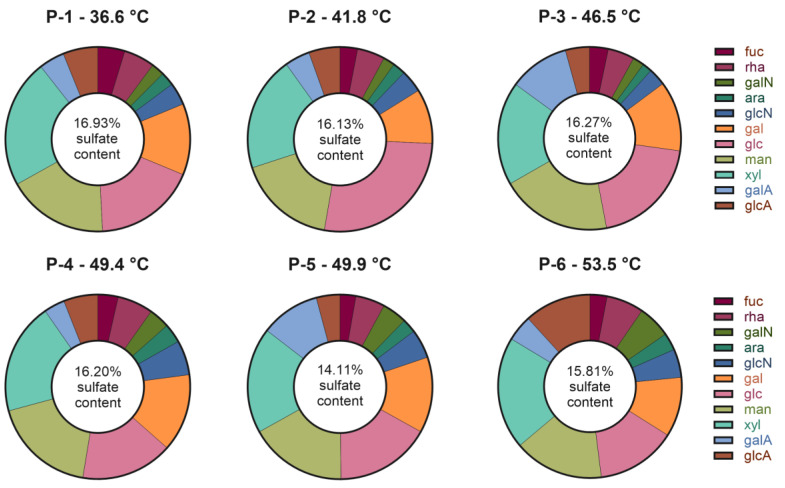
Monosaccharide composition and sulphate content of the six M-PSs extracted from mature muds, reported as molar percentages (mol%) and *w*/*w* percentages, respectively. Abbreviations: fuc, fucose; rha, rhamnose; galN, galactosamine; ara, arabinose; glcN, glucosamine; gal, galactose; glc, glucose; man, mannose; xyl, xylose; galA, galacturonic acid; glcA, glucuronic acid.

The analysis of monosaccharide composition of lyophilized samples revealed the presence of 11 different monosaccharide moieties, including two uronic acids and two amino sugars, as shown in Figure 4. Xylose, mannose and glucose were the most abundant monosaccharides, ranging between the 16–23% (mol%) in all the samples, except for the glucose in P-2 and P-6 (27 and 14%, respectively). Galactose ranged between 10–14%, while the content of the other monosaccharides was lower than 7%. Uronic acids and sulphate content, which confer the negative charge to the polymers, confirming zeta potential and FT-IR spectra analyses, ranged between 9.65 and 16.36% (mol%) and 14.11 and 16.93% (*w*/*w*), respectively. The exact monosaccharide composition quantification is reported in Table S3.

The percentage of sulphate groups was notably high, and this characteristic has been associated with various biological activities. Specifically, a strong immunomodulatory ability has been reported both in vitro and in vivo [33,34]. As discussed by Wang et al. [35], arabinose, xylose, mannose, and galactose are monosaccharides whose presence correlates with macrophage-stimulating activity within the broader context of immunomodulatory abilities. Furthermore, their review highlighted that the anti-inflammatory activity of polysaccharides is primarily influenced by the presence of sulphate, fucose, and galactose [35].

Moreover, the monosaccharide composition and the sulphate content of M-PSs obtained from mature mud samples, were very similar to the one obtained by Zampieri et al. [16] in the characterization of M-PS extracted from a therapeutic mud, matured at 39 °C. This result also highlights the homogeneity in monomers composition of samples derived from the top layer of mature muds, where polysaccharides are produced and modified. Additionally, it is worth noting that this monosaccharide composition seems very peculiar and unique. Compared to polysaccharides’ monomers composition of peloids from Mongolia [36] and Montenegro [37], Euganean M-PSs possess a very high number of different monosaccharides.

Finally, it’s intriguing to notice that the analysis of monosaccharides composition and sulphate abundance, as well as FT-IR spectra and zeta potential, showed a high similarity between M-PS samples, especially considering the differences in cyanobacteria population composition observed in the NGS microbiota analyses. This result could be explained considering the presence of a consortia of microorganisms that are able to produce and modify EPSs, including polysaccharides [38]. For example, Halary et al. [39], recently analysed the bacterial communities associated with cyanobacteria strains isolated from Thermal mud of Balaruc-les-Bains (France). Their findings suggest the presence of a potential metabolic association between cyanobacteria and heterotrophic bacteria within the so-called cyanosphere. This association is driven by the reciprocal exchange of common resources, including carbon and nitrogen sources [39]. Additionally, when comparing the monosaccharide composition of *Phormidium* sp. ETS-05 polysaccharides, as described by Zampieri and colleagues in 2020 [7] and also remarked in the first study on polysaccharides produced by the whole Euganean microbiota [16], we can observe a distinct composition in monomers. This observation underscores that, despite this species being the most abundant under certain temperature conditions, polysaccharides undergo various modifications and alterations that result in a homogeneous final product.

### 2.4. Anti-Inflammatory Potential of the Different M-PS

To assess the anti-inflammatory potential of the M-PSs extracted from mud maturated at different temperatures, we used a zebrafish model of chemical-induced inflammation based on exposure to copper sulphate pentahydrate (CuSO_4_·5H_2_O). This treatment generates oxidative stress and induces a systemic inflammation [40] that leads to a general developmental impairment of zebrafish larvae and a delay of processes like swim bladder insufflation and operculum bone ossification. As previously demonstrated [7,16], thanks to their therapeutic potential, PS administration can contrast this developmental delay. Moreover, the recovery can be directly evaluated by morphometric analyses of target traits.

The analyses were conducted after the induction of inflammation and the subsequent exposure of the larvae to the M-PS samples, object of this study, for different periods (18, 24 and 48 h). In this way we aimed to determine possible differences in the anti-inflammatory capabilities of M-PSs. The ossification of the operculum bone was analysed only after 24 and 48 h of exposure to M-PS to facilitate a correct evaluation of the differences between samples. Representative pictures of 5-dpf zebrafish larvae after 48 h of treatment (CTRL, CuSO_4_ and CuSO_4_ + M-PS) are reported in Figure S5.

**Figure 5:**
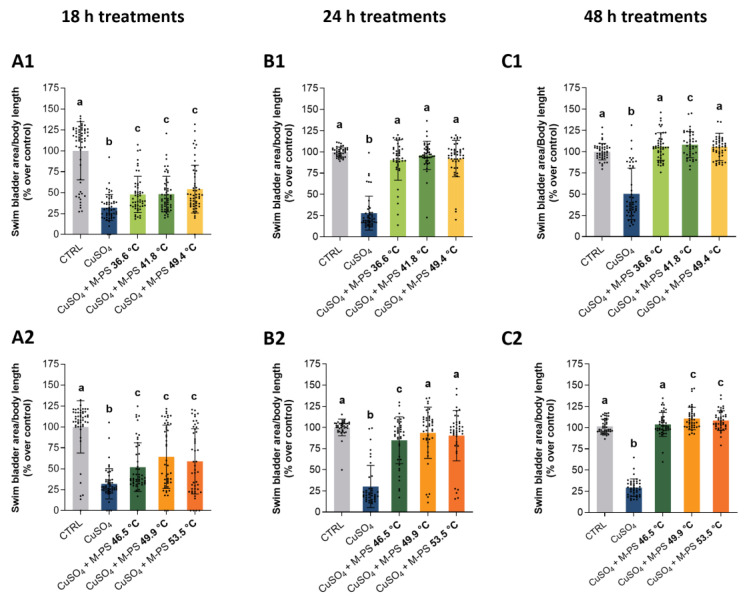
Recovering of normal developmental parameters of swim bladder area on larvae treated with M-PS after CuSO_4_·5H_2_O induced inflammation at 3 dpf. (**A1**,**A2**) Recovery from inflammation after 18 h of treatment with M-PSs extract from mud maturated at 36.6, 41.8, 49.4 °C (**A1**) and 46.5, 49.9, 53.5 °C (**A2**). (**B1**,**B2**) Recovery from inflammation after 24 h of treatment with M-PSs extract from mud maturated at 36.6, 41.8, 49.4 °C (**B1**) and 46.5, 49.9, 53.5 °C (**B2**). (**C1**,**C2**) Recovery from inflammation after 48 h of treatment with M-PSs extract from mud maturated at 36.6, 41.8, 49.4 °C (**C1**) and 46.5, 49.9, 53.5 °C (**C2**). Data are compared to control values. The swim bladder area is normalized over body length and indicated as percentages over control. Black bars represent the mean ± SD of three independent experiments conducted with 15–20 larvae per treatment. Statistical analysis was performed using GraphPad Prism 10 (Brown–Forsythe and Welch ANOVA test followed by Dunnett’s T3 multiple comparisons test with individual variances computed for each comparison). Statistical significance was set at *p* < 0.05 and the results of the multiple comparisons are shown with letters (different letters show differences among data). The exact adjusted *p* values are listed in Tables S4 and S5.

**Figure 6:**
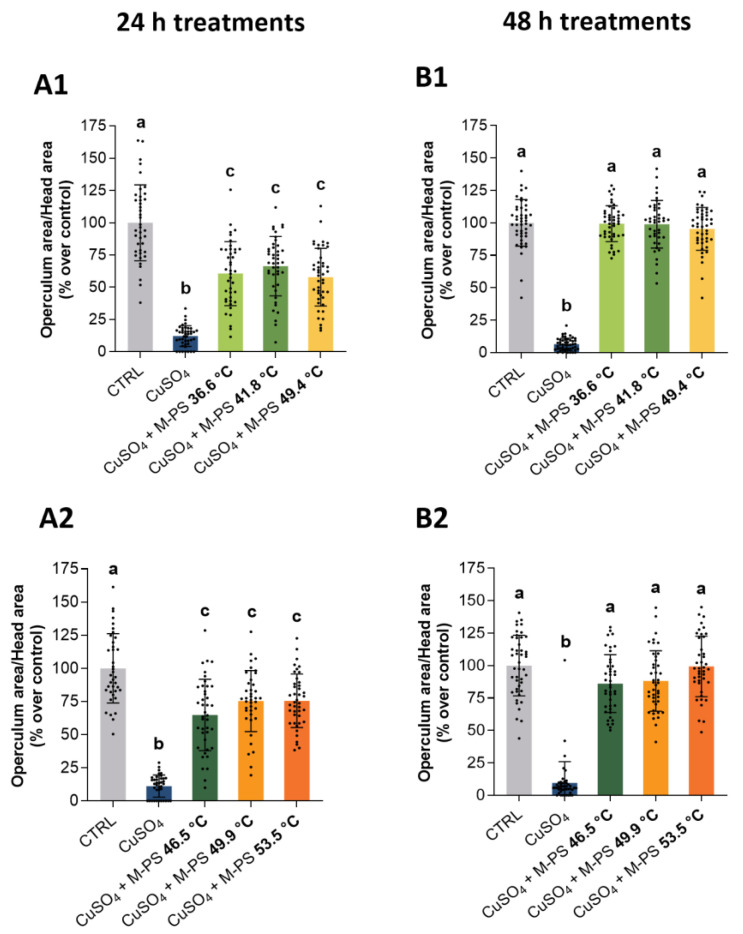
Recovering of normal developmental parameters of operculum bone area on larvae treated with M-PS after CuSO_4_·5H_2_O induced inflammation at 3 dpf. (**A1**,**A2**) Recovery from inflammation after 24 h of treatment with M-PSs extract from mud maturated at 36.6, 41.8, 49.4 °C (**A1**) and 46.5, 49.9, 53.5 °C (**A2**). (**B1**,**B2**) Recovery from inflammation after 48 h of treatment with M-PSs extract from mud maturated at 36.6, 41.8, 49.4 °C (**B1**) and 46.5, 49.9, 53.5 °C (**B2**). Data are compared to control values. The operculum bone area is normalized over total head area and indicated as percentages over control. Black bars represent the mean ± SD of three independent experiments conducted with 15–20 larvae per treatment. Statistical analysis was performed using GraphPad Prism 10 (Brown–Forsythe and Welch ANOVA test followed by Dunnett’s T3 multiple comparisons test with individual variances computed for each comparison). Statistical significance was set at *p* < 0.05 and the results of the multiple comparisons are shown with letters (different letters show differences among data). The exact adjusted *p* values are listed in Tables S6 and S7.

Surprisingly, at the end of treatment, swim bladder insufflation delay (Figure 5), operculum ossification (Figure 6), body length and eye area (Figures S6 and S7) were all similarly recovered to WT parameters or even better by all the used M-PSs. The temporal scan of the treatments showed a progression in the morphological traits rescue with a partial recovery at 18 and 24 h and a total recovery at 48 h. By contrast, the inflamed siblings, that were only exposed to fish water after the copper sulphate treatment, were not able to fully recover the developmental delay and showed only a small progressive recovery going from 18 h to 48 h post inflammation for the swim bladder insufflation (Figure 5) and a more reduced one for the operculum ossification (Figure 6). Similar results were obtained with the analysis of body length and eye area. Also in this case, at the end of M-PS treatment (48 h) significant differences were still present between only inflamed samples and WT or M-PS treated samples but not between WT and M-PS treated (Figures S6 and S7).

These results are in agreement with those obtained by Zampieri et al. [16] in which a single M-PS, extracted from a therapeutic mud at the end of a 39 °C maturation period, was tested.

The comparable recovery of morphometric traits by M-PSs, produced by microbial communities obtained at different temperatures, may be attributed to their similar chemical composition, identified through our chemical analyses. This result agrees with the notion, reviewed in Hou et al. (2020), that the anti-inflammatory properties of polysaccharides have close a relationship with their chemical structures, especially their monosaccharide compositions, molecular weights, chain conformations, glycosidic linkage types and positions and sulphate contents [41].

### 2.5. Locomotor Activity of Zebrafish Larvae after M-PS Treatments

Besides developmental delay, treatment with copper sulphate leads to a dysfunctional locomotor behaviour of zebrafish larvae [16,42,43]. We used the light-dark locomotion test to analyse the recovery from the swimming impairment after the inflammatory induction and the following treatment with M-PSs for 24 and 48 h.

**Figure 7:**
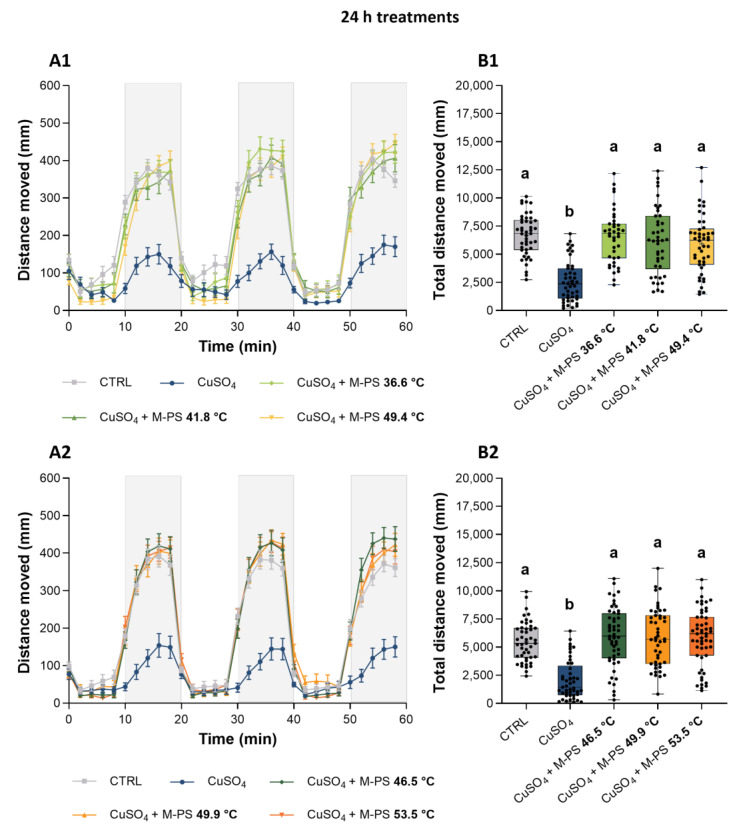
Analysis of locomotor activity of larvae treated with M-PS after CuSO_4_·5H_2_O inflammation at 3 dpf. (**A1**,**A2**) Recovery from inflammation after 24 h of treatment with M-PSs extract from mud maturated at 36.6, 41.8, 49.4 °C (**A1**) and 46.5, 49.9, 53.5 °C (**A2**). (**B1**,**B2**) Total distance moved by larvae depending on treatment. Lightning protocol: 10 min of light (white background) and 10 min of dark (grey background). Data were obtained considering 2 min intervals during the 60 min sessions. Black bars represent the mean ± SEM of three independent experiments conducted with 15–20 larvae per treatment in (**A1**,**A2**) and mean ± SD in (**B1**,**B2**). Statistical analysis was performed using GraphPad Prism 10 (Brown–Forsythe and Welch ANOVA test followed by Dunnett’s T3 multiple comparison test with individual variances computed for each comparison). Statistical significance was set at *p* < 0.05 and the results of the multiple comparisons are shown with letters (different letters show differences among data). The exact adjusted *p* values are listed in Tables S8 and S9.

**Figure 8:**
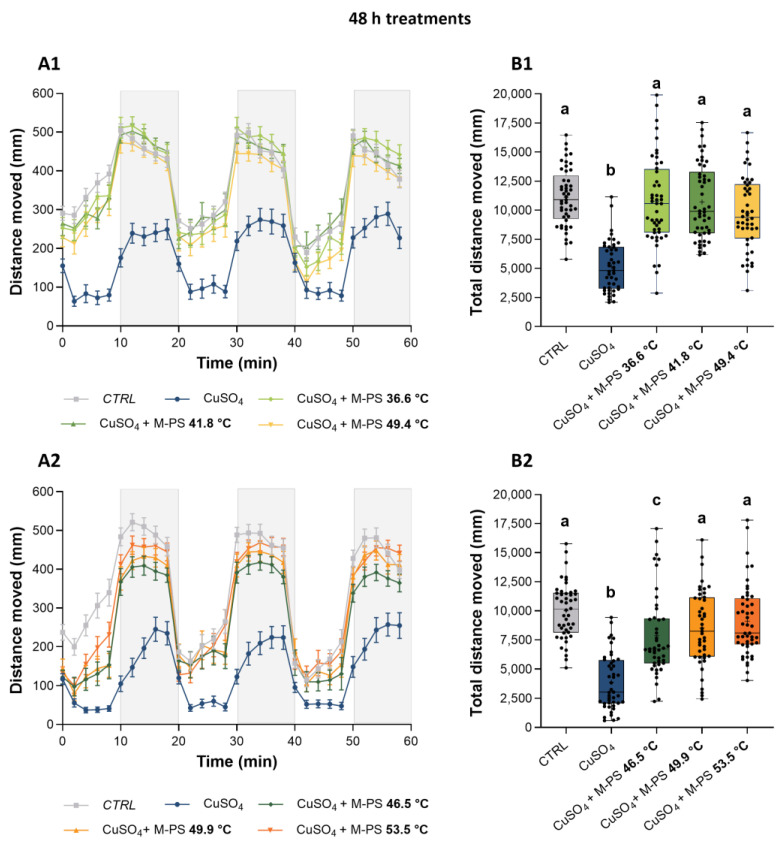
Analysis of locomotor activity of larvae treated with M-PS after CuSO_4_·5H_2_O inflammation at 3 dpf. (**A1**,**A2**) Recovery from inflammation after 48 h of treatment with M-PSs extract from mud maturated at 36.6, 41.8, 49.4 °C (**A1**) and 46.5, 49.9, 53.5 °C (**A2**). (**B1**,**B2**) Total distance moved by larvae depending on treatment. Lightning protocol: 10 min of light (white background) and 10 min of dark (grey background). Data were obtained considering 2 min intervals during the 60 min sessions. Black bars represent the mean ± SEM of three independent experiments conducted with 15–20 larvae per treatment in (**A1**,**A2**) and mean ± SD in (**B1**,**B2**). Statistical analysis was performed using GraphPad Prism 10 (Brown–Forsythe and Welch ANOVA test followed by Dunnett’s T3 multiple comparisons test with individual variances computed for each comparison). Statistical significance was set at *p* < 0.05 and the results of the multiple comparisons are shown with letters (different letters show differences among data). The exact adjusted *p* values are listed in Tables S10 and S11.

Briefly, the dark-to-light switch normally determines a rapid reduction in locomotor activity, while the light-to-dark change results in a rapid increase in motor activity [44]. Although this natural trend was present at both time points and in all samples, including only inflamed larvae, the latter showed a lower increase in locomotor activity, particularly during the dark phases (Figure 7(A1,A2) and Figure 8(A1,A2)). On the contrary, treatments with all the tested M-PSs, at both time points, allowed recovery of larvae swimming activity, as shown in Figure 7 (A1,A2) and Figure 8 (A1,A2), and also by the quantification of the total distance moved (Figure 7 (B1,B2) and Figure 8 (B1,B2)).

These results agree with our previous findings [16], related to the analysis of the anti-inflammatory potential of M-PS extracted from a mud maturated at 39 °C.

### 2.6. RT-qPCR Expression Analysis

As final support for the validation of the beneficial effects of M-PSs, the expression of genes linked to the inflammatory pathways was analysed by RT-qPCR on two representative M-PSs extracted from muds maturated at low (36.6 °C) and high (49.4 °C) temperatures. The transcription levels of *matrix metalloproteinases 9* (*mmp9*) [45], *serum and glucocorticoid regulated kinase 1* (*sgk1*) [46] and *suppressor of cytokine signaling 3a* (*socs3a*) [47] were significantly up-regulated in inflamed larvae when compared to control siblings (Figure S8), while the treatment with the M-PS samples significantly reduced their expression. These results further confirm that M-PSs, despite the maturation temperature, has the potential to reduce the inflammatory status.

## 3. Materials and Methods

### 3.1. Materials

*N*,*N*-dimethylformamide (227056), Na_2_EDTA (E5134), Copper sulphate solution 0.1 M (CuSO_4_·5H_2_O, 1027841000), Tricaine (MS222, A5040), 3-methyl cellulose (M0387) were purchased by Merk (Darmstadt, Germany). Membranes for dialysis (Spectrum™ Spectra/Por™, 12791138) were purchased from Fisher Scientific (Milan, Italy). Monosaccharides standards were purchased from Sigma Chemicals Co. (St. Luis, MO, USA), except for L(+)-rhamnose (Carlo Erba Reagents, Milan, Italy) and D-glucosamine (Alfa Aesar, Delphi, India).

### 3.2. Mud Sampling and Analysis of Chlorophyll and Microbial Content

Sampling of the mud was conducted in five spas of the Euganean Thermal District located in Abano Terme and Montegrotto Terme (Padova, Italy) in June 2022. The samples were collected from six concrete ponds filled with thermal water at different temperatures, as reported in Table 1. Briefly, for each sampling site, at the end of the maturation process, 20 sub-samples of 1–1.5 cm of the upper layer of the mud were collected using a sterile corer. Then, sub-samples belonging to the same pond were mixed in sterile containers to obtain a representative final homogeneous mature mud sample. Containers were maintained in a refrigerated box to rapidly reach the laboratory where mature mud samples were aliquoted and processed. We focused on studying the upper layer of mature mud because it is the primary site for the growth and development of cyanobacteria, which have been previously identified as a significant group of microorganisms responsible for conferring therapeutic properties to muds [12,13].

Samples from the microbial biofilm on the surface of the mature mud were collected with sterile pipettes and used for light and fluorescent microscopy analysis to define the cyanobacteria population traits as described in Gris et al. [9].

Five grams of each mud sample were dried at 60 °C for 24 h in the dark and powdered in a mortar. From each sample, three subsamples of 0.5 g were resuspended in *N*,*N*-dimethylformamide and incubated at 4 °C in the dark for 24 h. Samples were then centrifuged at 20,000× *g* for 5 min. Supernatants were analysed using a spectrophotometer (Cary Series ultraviolet visible, Agilent Technologies, Santa Clara, CA, USA), recording the absorption spectrum from 350 to 750 nm.

Chlorophyll *a* and carotenoids concentrations were determined according to the equations of Moran and Chamovitz [48,49].

### 3.3. DNA Extraction, Amplification and Sequencing

Three aliquots (1.5–2 g) of fresh mature mud samples from the six selected sites were stored at −20 °C for molecular analyses. The total genomic DNA was extracted from samples in biological duplicates using the DNeasy^®^ PowerSoil^®^ Pro Kit (QIAGEN GmbH, Hilden, Germany) following the manufacturer’s instructions. The DNA quantification and quality assessment were performed using NanoDrop (ThermoFisher Scientific, Waltham, MA, USA).

Microbial composition was determined by amplifying the hypervariable V4 region of bacterial and archaeal 16S rRNA gene, using forward and reverse degenerate primers 515F-806R [50]. Amplicons were purified through AMPure XP beads (Beckman Coulter, Brea, CA, USA), libraries were prepared with Illumina DNA Prep with IDT for Illumina DNA/RNA UD Indexes Set C (Illumina Inc., San Diego, CA, USA). Sequencing was then performed with Illumina NovaSeq 6000 platform (2 × 150 bp, paired-end). The raw reads were deposited in the sequence read archive database (SRA) of NCBI under the BioProject PRJNA624369.

### 3.4. Sequencing Data Analysis

The taxonomic assignment was conducted using a custom-made pipeline employing the dada2 package in R 4.3.1, which generated a list of unique sequences from the V4 region identified in the sample, called ASVs (Amplicon Sequence Variants). These ASVs were then assigned to a specific taxonomic group using the SILVA 138.1 database as a reference (https://www.arb-silva.de/, the database was downloaded on 9 May 2023). Subsequently, the NGS results were analysed following the dada2 amplicon analysis procedure. The most abundant ASVs (relative abundance > 0.01%) were grouped by Phyla to get an overview of the microbiota composition. Then, ASVs assigned to Cyanobacteria phylum (with a relative abundance higher than 0.01% in all samples) were selected and manually curated and compared with sequences in the GenBank database (https://www.ncbi.nlm.nih.gov/, accessed on 8 March 2023), using BLAST similarity search (nucleotide collection and 16S ribosomal RNA sequences database), in order to validate the results. To assign an univocal ETS (Euganean Thermal Springs) code we compared the ASVs with the OTUs obtained by Gris et al. [9].

### 3.5. Extraction of Polysaccharides from Mature Muds

Fresh mature muds were centrifuged at 5000× *g* for 20 min at room temperature. Each pellet, constituted by the mature mud sampled in a specific sampling site, was divided into two subsamples of 10 g. For each subsample, Na_2_EDTA 0.1 M was added and mixed overnight using a rocker. The mixtures obtained were centrifuged at 5000× *g* for 20 min at room temperature and the supernatants were collected in a tube. This process was repeated three times, reducing Na_2_EDTA exposure time to 1 h [51]. Supernatant containing polysaccharides was dialyzed for 72 h at 4 °C using a 3.5 kDa cut-off membrane and lyophilized in a freeze drier. The carbohydrates concentration in the samples resuspended in bi-distilled water were quantified using the Dubois method [52]. The lyophilized compounds, extracted from the mature muds, were then named Microbial PolySaccharides and shortened to M-PS. The different M-PSs used for the following tests are identified by the maturation temperature of the mud from which they were extracted (e.g., M-PS 36.6 °C). The total polysaccharides content was measured also directly on the lyophilized mature mud powders, according to Safarik and Santruckova [53].

### 3.6. Characterization of Microbial Polysaccharides

FT-IR spectrum was collected using a Bruker Alpha FTIR spectrometer (Bruker, Billerica, MA, USA). M-PS samples were ground into powder with dry KBr in the ratio 1:100 and then pressed into pellets. Absorbance spectra were measured over the wavenumber range from 4000 to 400 cm^−1^ with a resolution of 1.42 cm^−1^.

The zeta potential of M-PS at a concentration of 5 mg/mL in deionized water was recorded using a Zetasizer Nano ZS (Malvern Instruments Limited, Worcestershire, UK). The measurements were performed in triplicate at 25 °C with the instrument in automatic mode (minimum of 10 to a maximum of 100 runs for each measurement). Each sample was determined with three independent measurements and the average value was taken.

To determine the monosaccharide composition, lyophilized M-PS were firstly hydrolysed with 2N trifluoroacetic acid (TFA) at 120 °C for 120 min. The hydrolysates were evaporated by a rotary evaporator (Rotavapor R-100, Büchi, Flawil, Switzerland) to remove TFA and resuspended in 2 mL of deionized water before being analysed adopting a Dionex ICS-2500 ion exchange chromatograph (Dionex, Sunnyvale, CA, USA) equipped with an ED50 pulsed amperometric detector operating with a gold working electrode and a DionexCarboPac PA1 column of 250 mm length and 4.6 mm internal diameter (Thermo Scientific, Waltham, MA, USA). Eluents used were HPLC–grade water (A), 0.185 M sodium hydroxide (B), and 0.488 M sodium acetate (C). In the first stage of the analysis (from injection time to 20 min), the eluent was constituted by 90% A and 10% B; in the second stage (from 20 to 30 min), the eluent was constituted by 50% B and 50% C; in the final stage (from 30 to 60 min), the eluent was constituted by 90% A and 10% B. The flow rate was kept at 1 mL/min. Peaks for each sugar were identified and quantified on the basis of known standards. Data are expressed as molar ratios (%).

For sulphate analysis, the same Dionex ICS-2500 ion exchange chromatograph (Dionex,, Sunnyvale, CA, USA) system was equipped with a continuously regenerated anion-trap column, a continuous anionic self-regenerating suppressor, a conductivity detector (ED50), an IonPac PA11 4 _ 250 mm column (Dionex, Sunnyvale, CA, USA), and a reagent-free Dionex system producing high-purity 50 mM KOH at a flow rate of 2 mL/min. Sulfate solutions (1 to 10 mg/L; Fluka, Buchs, Switzerland) were used as standards.

### 3.7. Zebrafish Maintenance

Zebrafish were maintained and reared following standard guidelines [54]. Zebrafish embryos were obtained by natural spawning and raised in fish water in Petri dishes at 28 °C. Developmental stages were expressed in hours and days post fertilization (hpf, dpf). All experiments described in this paper were performed on larvae before the free-feeding stage and thus did not fall under animal experimentation laws according to EU Animal Protection Directive 2010/63/EU. No adult zebrafish were sacrificed for this study. Larvae were anaesthetized or euthanized with fish water containing 0.16 or 0.3 mg/mL of tricaine, respectively. In this work, only Wild Type (WT) larvae were used.

### 3.8. Chemical Inflammation Induction

3-dpf larvae were used as the starting point for each experiment. A copper sulphate solution (CuSO_4_·5H_2_O) was prepared in fish water just before the start of each experiment. Larvae were exposed to 20 μM copper sulphate solution for two and a half hours, followed by several washings and medium changes with fish water to eliminate potential residuals of the compound. M-PS at 50 μg/mL concentration were given after the inflammation induction for different time intervals according to the specific experiment (18–24–48 h for the morphological and locomotor activity analyses and 6 h for the gene expression analyses). This concentration was selected on the basis of our previous works [7,16]. Specifically, the experiments were divided into two sample sets and, in each set, we compared the effectiveness of 3 different M-PSs (M-PSs 36.6, 41.8, 49.4 °C and 46.5, 49.9, 53.5 °C) for the recovery of the morphological traits and the behaviour evaluation. For the RT-qPCR analyses only two M-PSs obtained by mud matured at 36.6 and 49.4 °C were used. Each experiment was performed three times with 15–20 larvae per replica.

### 3.9. Morphological Traits Analysis and Image Processing

Larval morphology was analysed after inflammation induction and treatment with M-PSs for 18, 24 and 48 h, taking into account the following traits: body length (snout to tail), eyes area (measured as the area of a polygon), operculum bone ossification and swim bladder insufflation. The morphological traits analysed are graphically represented in Figure S5A. Operculum bone size was visualised by Alizarin red S staining and measured according to Tarasco et al. [55] and as previously reported [7,16]. The swim bladder area was normalized for the body length and the operculum area for the head area [7,16]. Larvae were anaesthetized with tricaine and arranged sideways in 3% methyl cellulose in microscope slides. ImageJ software v1.52a was used to compare treated larvae and controls on digital micrographs taken with a Leica M165 FC stereoscopic microscope (Leica Microsystems, Milan, Italy) equipped with a Leica DFC7000 T digital camera (Leica Microsystems, Milan, Italy).

### 3.10. Behaviour Analysis

Larval behaviour and locomotion were evaluated after copper sulphate inflammation induction and M-PSs treatment. Larvae that received the inflammatory stress were either exposed to M-PSs for 24 or 48 h or to fish water (positive control). Larvae were positioned individually in 48 multi-well plates with 0.9 mL of sterile fish water. Movement of every larva was recorded using the DanioVision Observation Chamber (Noldus, Wageningen, The Netherlands). The chamber temperature was maintained at 28 °C for the length of the analysis. Larvae were pre-adapted for 10 min in dark and then exposed to three alternating light/dark cycles of 10 min. EthoVision X.T 8.5.614 video tracking software (Noldus, Wageningen, The Netherlands) was used to assess the distance moved every 2 min. Total distance moved was calculated summing the distance travelled by each larva.

### 3.11. RNA Isolation, cDNA Synthesis and Expression Analysis

For expression analysis, total RNA was extracted from pools of 15 3-dpf larvae with TRIzol reagent (15596018, Thermo Fisher Scientific), Milan, Italy). Poly(A) mRNA was purified from 3.5 μg of total RNA with Dynabeads “mRNA direct kit” (61011, Thermo Fisher Scientific, Milan, Italy) and used for cDNA synthesis with the High-Capacity cDNA Reverse Transcription Kit (4368813, Thermo Fisher Scientific, Milan, Italy) according to the manufacture’s protocol. Real time qPCR was performed in triplicate with the SYBR green method using the CFX384 Touch Real-Time PCR Detection System (Bio-Rad Laboratories, Hercules, CA, USA) and the 5× HOT FIREpol EvaGreen qPCR Mix Plus (08-24-0000S, Solis BioDyne, Tartu, Estonia). Data were normalized to the expression of *gapdh* to standardize the results by eliminating variations in mRNA and cDNA quantity and quality. The amplification protocol was 95 °C for 12 min, followed by 40 cycles at 95 °C for 30 s, 60 °C for 30 s and 72 °C for 30 s. Results were analysed according to the ΔΔCt method using the Bio-Rad CFX Manager software Version 3.1 (Bio-Rad Laboratories, Hercules, CA, USA). Primer sequences are reported in Table S12.

### 3.12. Statistical Analysis

Statistical analysis was performed using Graph Pad Prism (Version 10.1.2, GraphPad software). The statistical analysis of comparison between control and treated samples was performed with one-way ANOVA followed by Tukey’s multiple comparison test or Brown-Forsythe and Welch ANOVA tests followed by Dunnett’s multiple comparison test with individual variances computed for each comparison. Data are presented as means ± SD (means ± SEM in Figure 7(A1,A2) and Figure 8(A1,A2)). The *p*-values were indicated with letters that show the results of the multiple comparison test in all figures or with the following symbols in Figure S8: * *p* < 0.05, ** *p* < 0.01, *** *p* ≤ 0.001 (significance was set at *p <* 0.05). Experiments were conducted at least three times with biological replicas composed by 15–20 larvae.

## 4. Conclusions

The objective of this study was to investigate whether heteropolysaccharides extracted from various mature muds of Euganean Thermal District’s spas exhibit differences in their anti-inflammatory capabilities.

The beneficial properties of M-PS extracted from a therapeutic mud produced at 39 °C have already been explored, revealing that polysaccharides produced by the whole microbial community possess antioxidant and anti-inflammatory activity in vivo [16]. However, until now, no studies have investigated the therapeutic effects of biomolecules extracted from mature muds obtained not following the traditional mud maturation process in a strict manner.

The analysis of the microbiota composition present in the mud samples matured at different temperatures (ranging between 36.6 °C and 53.5 °C) revealed a relatively stable microbial community at phylum level across the temperature range studied. Yet, big differences in cyanobacteria species relative abundances were observed with increasing temperatures. Despite the varying composition of cyanobacteria, the chemical characterization of the M-PSs extracted from these muds demonstrated the presence of a strong conservation in the chemical composition, as shown by similar FT-IR spectra, zeta potential and monosaccharides and sulphate groups abundance.

In parallel, we analysed the anti-inflammatory potential of these M-PSs using a zebrafish inflammation model. The analysis of the recovery of developmental delay and locomotor behaviour, after the treatment with M-PSs on inflamed zebrafish larvae, showed a similar beneficial potential. These results have been confirmed by expression analysis on zebrafish larvae treated with two representative M-PSs, showing the downregulation of genes associated with inflammation.

These findings can be ascribed to the chemical homogeneity of the extracted M-PSs, possibly due to a concerted action of the complex microbiota community that colonize the mud in the modification of EPSs during the maturation process.

The work described in this paper supports the efficacy of the Euganean Thermal muds for the treatment of chronic inflammation and the presence of a homogeneous beneficial product among the different spas of the area.

## Figures and Tables

**Table 1:** Average values of physico-chemical parameters (n = 3) of water samples from the selected thermal spas in Abano and Montegrotto Terme. Sampling was performed in June 2022. In all spas, mud maturation was performed in ponds. Protective roof indicates the presence of structures protecting ponds from solar radiation (YES, NO, Y/N indicates partial coverage). Chlorophyll (Chl *a*) and carotenoid (Car) values are presented as media ± standard deviation. Other abbreviations: total dissolved solids (TDS), identification number (ID).

Sampling Site ID	Spa Location	Temperature (°C)	pH	TDS (ppm)	Protective Roof	Chl *a* (µg/g_MUD_)	Car (µg/g_MUD_)
P-1	Abano	36.6	6.15	2790	YES	19.0 ± 1.8	6.7 ± 0.5
P-2	Montegrotto	41.8	7.15	2920	Y/N	8.7 ± 0.2	4.0 ± 0.1
P-3	Montegrotto	46.5	6.20	3000	YES	17.6 ± 0.2	7.8 ± 0.0
P-4	Montegrotto	49.4	6.90	3000	Y/N	20.3 ± 1.5	9.3 ± 0.5
P-5	Montegrotto	49.9	6.27	1840	YES	16.5 ± 0.6	6.4 ± 0.2
P-6	Montegrotto	53.5	6.09	3000	Y/N	14.1 ± 0.4	7.3 ± 0.3

**Table 2:** Average values ± standard deviation of zeta potential of M-PS samples from thermal spas in Abano and Montegrotto Terme in 2022. The lyophilized powder was dissolved in deionized water at a concentration of 5 mg/mL.

Sampling Site ID	Temperature (°C)	Zeta potential (mV)
P-1	36.6	−23.17 ± 1.46
P-2	41.8	−19.83 ± 1.50
P-3	46.5	−22.57 ± 1.83
P-4	49.4	−21.33 ± 1.25
P-5	49.9	−22.80 ± 1.71
P-6	53.5	−21.83 ± 0.74

## Data Availability

The raw read of the mud’s microbiota NGS sequencing have been deposited in the sequence read archive database (SRA) of NCBI under the BioProject PRJNA624369 (https://www.ncbi.nlm.nih.gov/bioproject/PRJNA624369/ accessed on 1 March 2024).

## Supplementary Materials

The following supporting information can be downloaded at: https://www.mdpi.com/article/10.3390/ijms25094999/s1.
