# Supplementary material for: Microbial Polysaccharides Extracted from Different Mature Muds of the Euganean Thermal District Show Similar Anti-Inflammatory Activity In Vivo
Source: Int J Mol Sci. 2024 May 3;25(9):4999. doi: 10.3390/ijms25094999 (PMC11084611; doi:10.3390/ijms25094999)
Supplement: Supplementary file 1 [file ijms-25-04999-s001.zip › Caichiolo-2024_supplementary materials.pdf]

Microbial Polysaccharides Extracted from Different Mature Muds of the Euganean Thermal District Show Similar Anti-Inflammatory Activity In Vivo

Micol Caichiolo [1], Raffaella Margherita Zampieri [1,2], Alessandra Adessi [3], Matilde Ciani [3], Fabrizio Caldara [4], Luisa Dalla Valle [1,*,†] and Nicoletta La Rocca [1,†]

[1] Department of Biology, University of Padova, Via U. Bassi 58/b, 35131 Padova, Italy; micol.caichiolo@phd.unipd.it (M.C.), raffaellamargherita.zampieri@phd.unipd.it (R.M.Z.), nicoletta.larocca@unipd.it (N.L.R.)

[2] Research Institute on Terrestrial Ecosystems (IRET), National Research Council (CNR), Via Madonna del Piano 10,50019 Firenze, Italy

[3] Department of Agriculture, Food, Environment and Forest (DAGRI), University of Florence, Via Maragliano 77, 50144 Firenze, Italy; alessandra.adessi@unifi.it (A.A.), matilde.ciani@unifi.it (M.C.)

[4] Pietro D'Abano Thermal Studies Center, Via Jappelli 5, 35031, Padova, Italy; fabrizio.caldara@centrostuditermali.org

* Correspondence: luisa.dallavalle@unipd.it; Tel.: +39-04-9827-6188

† These authors contributed equally to the work.

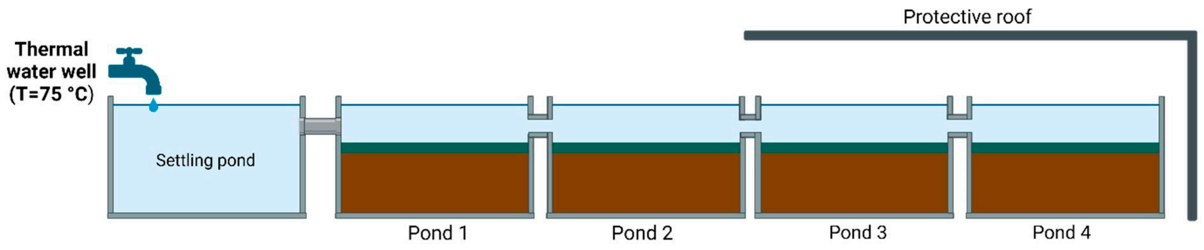

Figure S1: Representation of a typical pond system. Image created with Biorender.com.

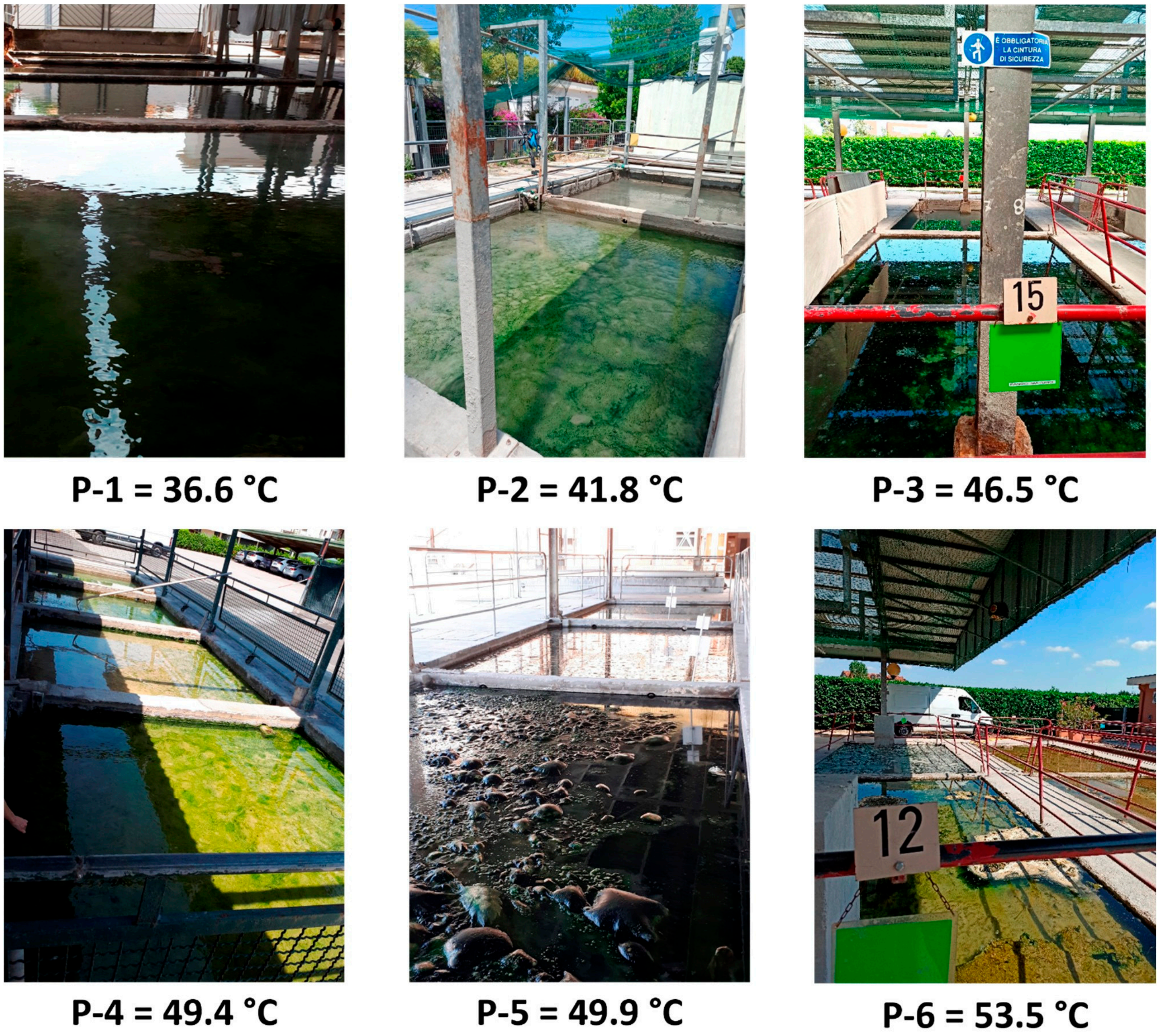

Figure S2: Pictures of the thermal environment of the sampled ponds.

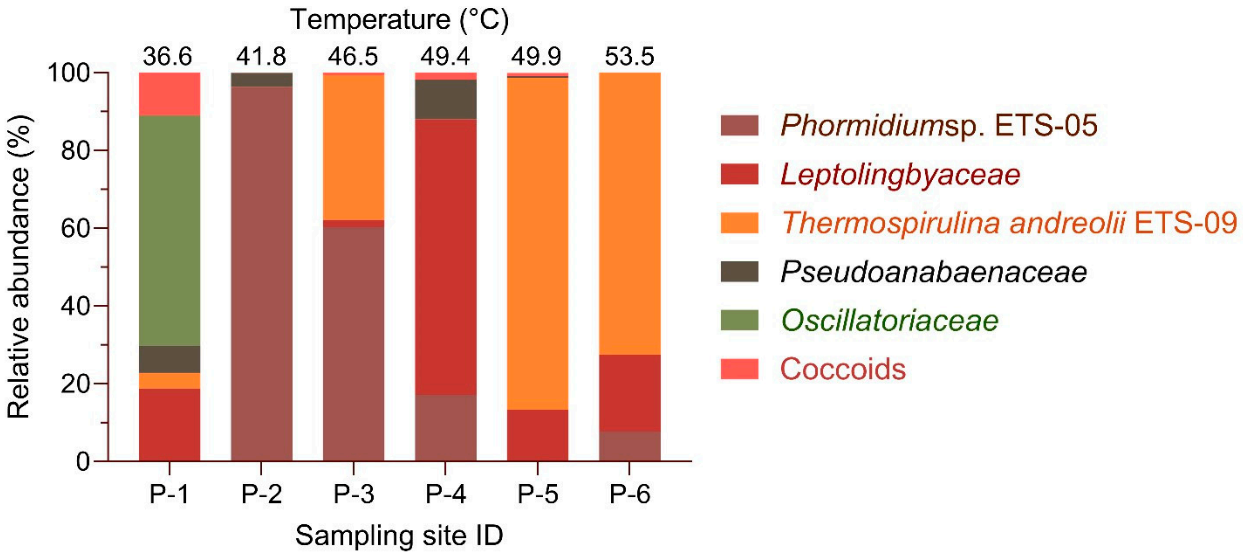

Figure S3: Cyanobacteria population composition of the collected samples, obtained referring the calculated volumes of each mophogenera to the total Cyanobacteria volume, as described in Gris et al., 2020 (see main text). Briefly, two replicates of each collected biofilm sample were diluted with thermal water (1:20, *v*/*v*) and 40 μl of formalin was added. Using a 40X objective, about 40 fields were analysed and images were acquired using both visible and fluorescent light (Leica DM6 B; Leica, Wetzlar, Germany). Images were then analysed using ImageJ software, considering filaments as cylinders and dividing coccoid species in groups based on cell size.

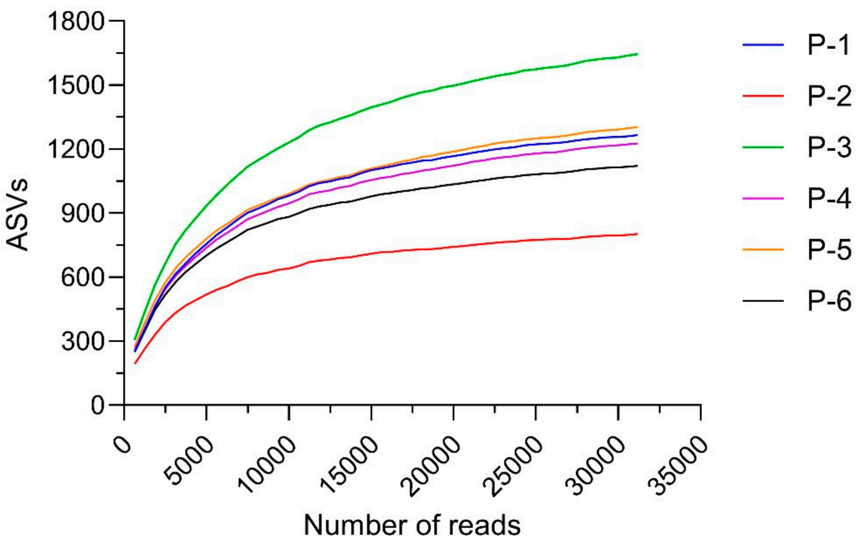

Figure S4: Average rarefaction curves (n=2) of each sample.

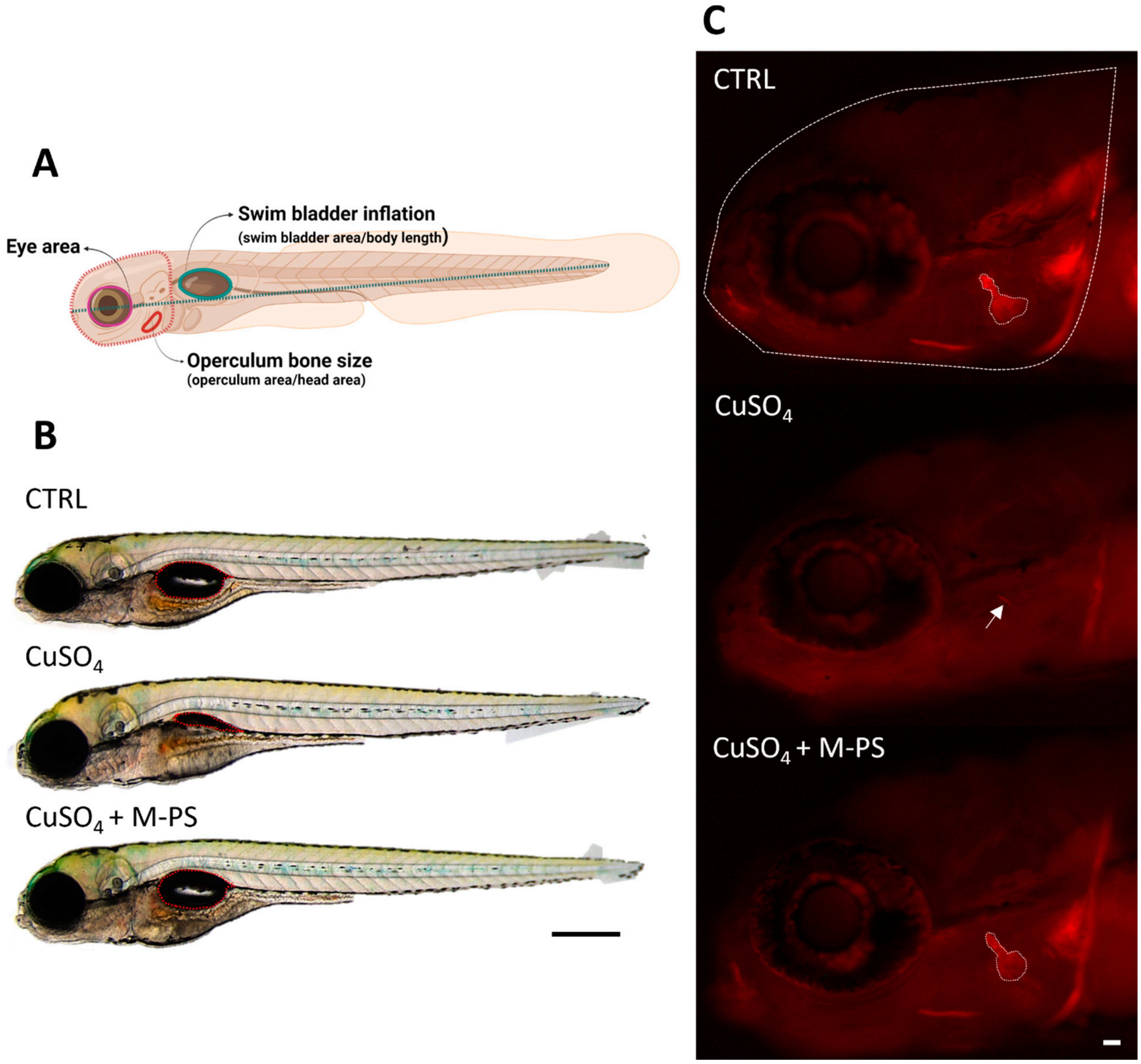

Figure S5: Representation of analysed morphological traits in zebrafish larvae. (A) Scheme representing the morphometric traits analysed on zebrafish larvae (Image created with Biorender.com). (B) Micrographs showing reduced inflation of swim bladder due to $CuSO_4 \cdot 5H_2O$ exposure ($CuSO_4$) and rescue of the inflation after 48 h M-PS treatment ($CuSO_4$ + M-PS). Sibling larvae (CTRL) are shown for comparison. Swim bladder is highlighted by red dotted lines. Scale bar = 500 µm. (C) Picture of 5-dpf larvae's head, stained with alizarin red S and showing the head and the operculum area evaluated though morphometric analyses. The operculum is highlighted by white dotted lines or white arrow. The micrographs show reduced operculum ossification after $CuSO_4 \cdot 5H_2O$ exposure ($CuSO_4$) and ossification rescue due to 48 h M-PS treatment ($CuSO_4$ + M-PS). Sibling larvae (CTRL) are shown for comparison. Scale bar = 50 µm.

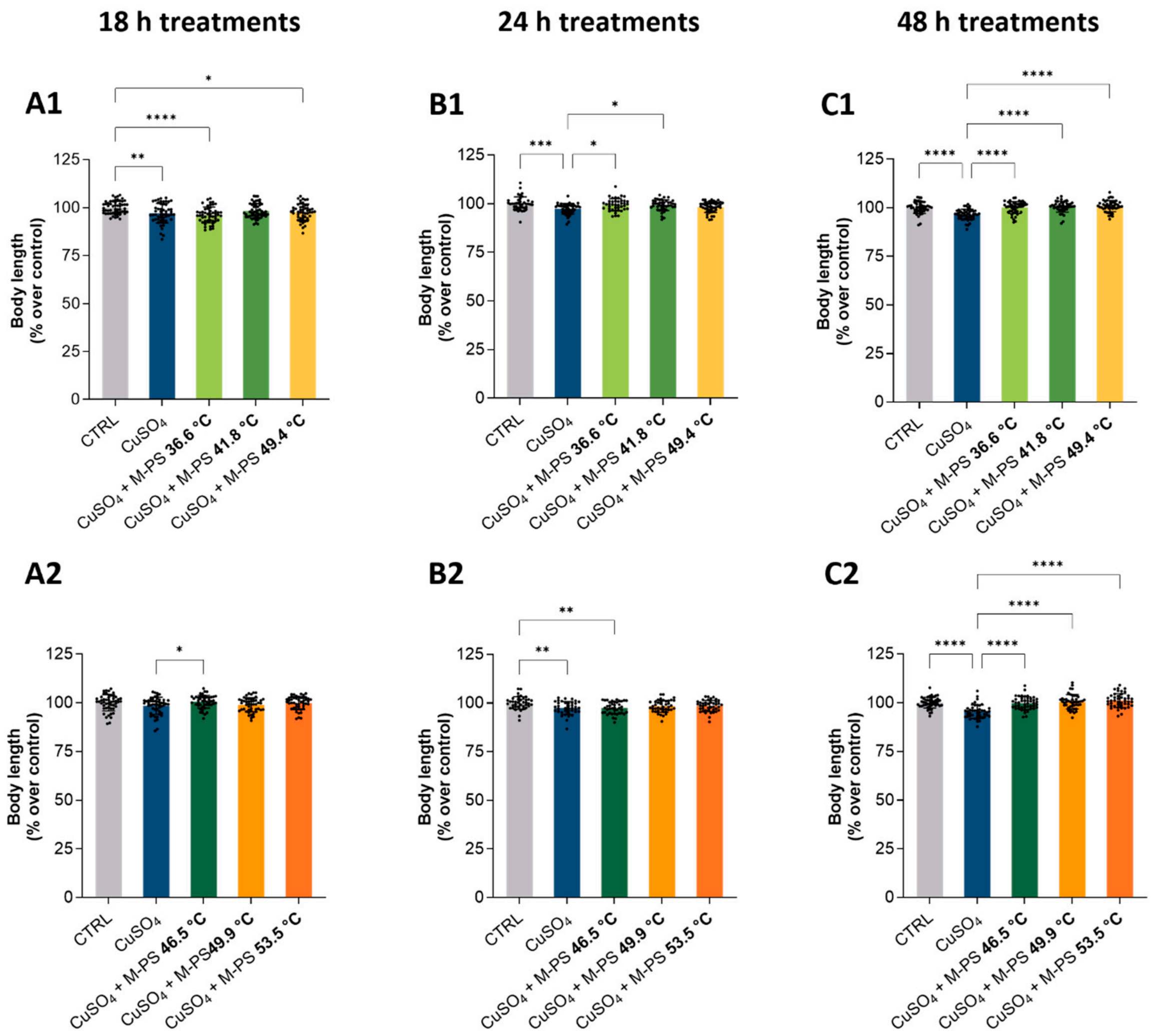

Figure S6: Analysis of body length on larvae treated with M-PS after $CuSO_4 \cdot 5H_2O$ inflammation at 3 dpf. (**A1-A2**) Recovery from inflammation after 18 hours of treatment with M-PS extract from mud maturated at 36.6, 41.8, 49.4 °C (A1) and 46.5, 49.9, 53.5 °C (A2). (**B1-B2**) Recovery from inflammation after 24 hours of treatment with M-PS extract from mud maturated at 36.6, 41.8, 49.4 °C (B1) and 46.5, 49.9, 53.5 °C (B2). (**C1-C2**) Recovery from inflammation after 48 hours of treatment with M-PS extract from mud maturated at 36.6, 41.8, 49.4 °C (C1) and 46.5, 49.9, 53.5 °C (C2). Data are compared to control values and indicated as percentages over control. Black bars represent the mean ± SD of three independent experiments conducted with 15-20 larvae per treatment. Statistical analysis was performed using GraphPad Prism 10 (ordinary oneway ANOVA followed by Tukey's multiple comparisons test with a single pooled variance in A2-B1-B2-C1-C2 and BrownForsythe and Welch ANOVA test followed by Dunnett's T3 multiple comparison test with individual variances computed for each comparison in A1). Statistical significance: $^{*}p < 0.05$, $^{**}p < 0.01$, $^{***}p \leq 0.001$, $^{****}p \leq 0.0001$.

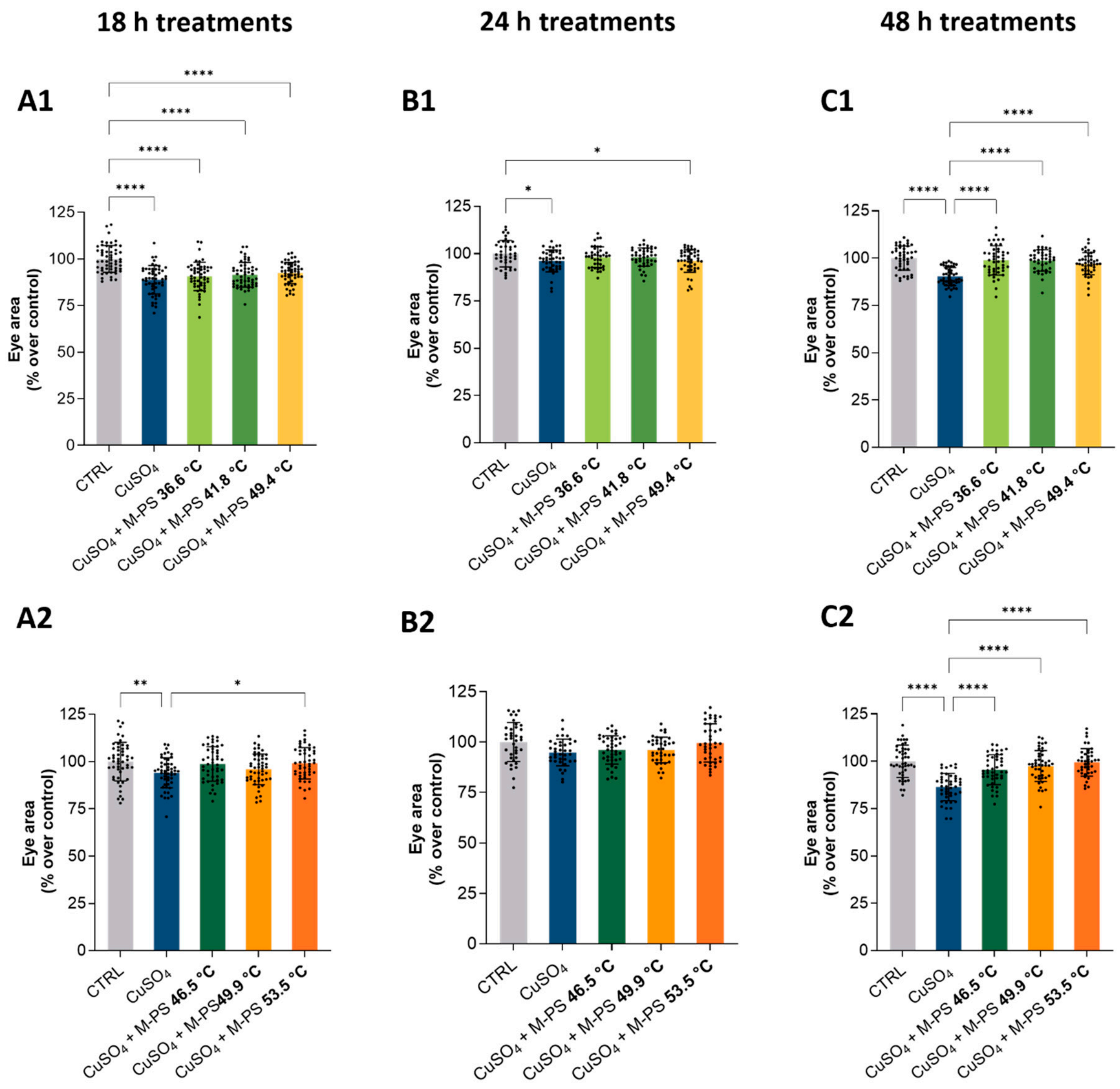

Figure S7: Analysis of eye area on larvae treated with M-PS after $CuSO_4 \cdot 5H_2O$ inflammation at 3 dpf. (A1-A2) Recovery from inflammation after 18 hours of treatment with M-PS extract from mud maturated at 36.6, 41.8, 49.4 °C (A1) and 46.5, 49.9, 53.5 °C (A2). (B1-B2) Recovery from inflammation after 24 hours of treatment with M-PS extract from mud maturated at 36.6, 41.8, 49.4 °C (B1) and 46.5, 49.9, 53.5 °C (B2). (C1-C2) Recovery from inflammation after 48 hours of treatment with M-PS extract from mud maturated at 36.6, 41.8, 49.4 °C (C1) and 46.5, 49.9, 53.5 °C (C2). The area was measured as the area of a polygon. Data are compared to control values and indicated as percentages over control. Black bars represent the mean ± SD of three independent experiments conducted with 15-20 larvae per treatment. Statistical analysis was performed using GraphPad Prism 10 (ordinary one-way ANOVA followed by Tukey's multiple comparisons test with a single pooled variance in A1-A2-B1-C2 and Brown-Forsythe and Welch ANOVA test followed by Dunnett's T3 multiple comparison test with individual variances computed for each comparison in C1). Statistical significance: *$p < 0.05$, **$p < 0.01$, ***$p \leq 0.001$, ****$p \leq 0.0001$.

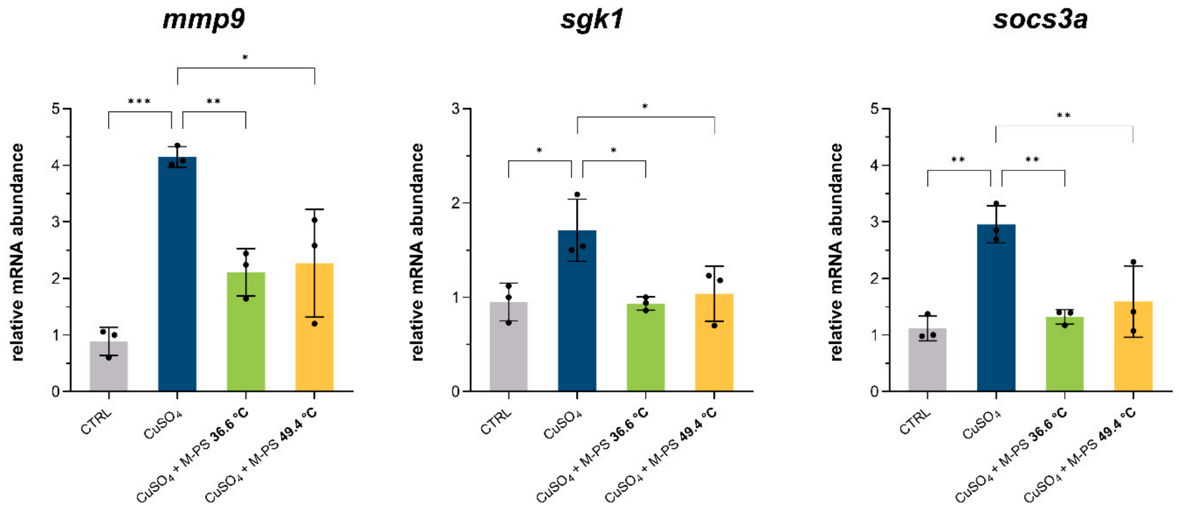

Figure S8: Relative mRNA abundance of genes involved in inflammatory response, analysed in 3-dpf zebrafish larvae after $CuSO_4 \cdot 5H_2O$ exposure, with and without M-PS treatment. M-PS were extracted from mud matured at 36.6 and 49.4 °C. The data represents the mean ± SD of three independent experiments conducted with 15-20 larvae. Statistical analysis was performed using GraphPad Prism 10 (ordinary one-way ANOVA followed by Tukey's multiple comparisons test with a single pooled variance). Statistical significance: *$p < 0.05$, **$p < 0.01$, ***$p \leq 0.001$.

Table S1: The Excel file contains a list of all the 16S rRNA sequences of Amplicon Sequence Variants (ASVs) obtained from the NGS analysis. Each mature mud sample was analysed in duplicate, so the file names are the following: P-1: A66-A67; P-4: A68-69; P-2: A70-A71; P-5: A72-A73; P-6: A74-75; P-3: A76-A77. Additionally, the spreadsheet includes ASVs corresponding to the most abundant taxa and Cyanobacteria (with abundances higher than 0.01%). Taxa are grouped based on the Phyla assigned by the pipeline.

Table S2: Average values of polysaccharides content ± standard deviation of extracted M-PS samples and of lyophilized mature muds from thermal Spas in Abano and Montegrotto Terme in 2022. Sugar content was quantified using the Dubois method.

Sampling site ID	Temperature (°C)	Sugar content in extracted M-PS (µg/g_{MUD})	Sugar content in mature mud (mg/g_{MUD})
P-1	36.6	54.02 ± 5.63	2.80 ± 0.27
P-2	41.8	43.24 ± 4.70	2.52 ± 0.20
P-3	46.5	72.02 ± 29.27	3.35 ± 0.84
P-4	49.4	76.10 ± 19.26	6.31 ± 1.04
P-5	49.9	58.06 ± 3.88	3.17 ± 0.87
P-6	53.5	75.27 ± 29.93	2.74 ± 0.25

Table S3: Monosaccharide composition quantification, as molar %, of M-PS from the six mature muds. Abbreviations: fuc, fucose; rha, rhamnose; galN, galactosamine; ara, arabinose; glcN, glucosamine; gal, galactose; glc, glucose; man, mannose; xyl, xylose; galA, galacturonic acid; glcA, glucuronic acid.

Monosaccharides (mol %):	P-1	P-2	P-3	P-4	P-5	P-6
fuc	4.73	3.06	3.29	3.56	2.76	3.00
rha	5.43	4.84	4.66	6.25	5.14	6.58
galN	2.26	2.03	2.03	3.64	4.34	5.78
ara	2.42	2.23	1.68	3.39	2.47	3.06
glcN	3.94	3.97	3.15	6.07	5.09	5.01
gal	12.50	9.59	12.38	13.60	13.28	10.52
glc	17.91	27.02	19.80	16.10	16.77	14.07
man	17.71	17.27	19.68	18.18	17.06	15.82
xyl	22.52	20.10	18.34	19.56	18.55	19.80
galA	4.49	4.41	10.71	3.66	10.39	4.60
glcA	6.10	5.48	4.27	5.99	4.15	11.76

Table S4: Exact adjusted P value resulted from Brown-Forsythe and Welch ANOVA test followed by post hoc Dunnett's T3 multiple comparison test of graphs A1-B1-C1 of Figure 5 (Analysis of swim bladder area).

Dunnett's T3 multiple comparisons test	Adjusted P Value A1	Adjusted P Value B1	Adjusted P Value C1
CTRL vs. CuSO4	<0.0001	<0.0001	<0.0001
CTRL vs. CuSO4 + M-PS 36.6 °C	<0.0001	0.1374	0.2772
CTRL vs. CuSO4 + M-PS 41.8 °C	<0.0001	0.7660	0.0395
CTRL vs. CuSO4 + M-PS 49.4 °C	<0.0001	0.2017	0.3848
CuSO4 vs. CuSO4 + M-PS 36.6 °C	0.0006	<0.0001	<0.0001
CuSO4 vs. CuSO4 + M-PS 41.8 °C	0.0003	<0.0001	<0.0001
CuSO4 vs. CuSO4 + M-PS 49.4 °C	<0.0001	<0.0001	<0.0001
CuSO4 + M-PS 36.6 °C vs. CuSO4 + M-PS 41.8 °C	>0.9999	0.9011	0.9993
CuSO4 + M-PS 36.6 °C vs. CuSO4 + M-PS 49.4 °C	0.8954	>0.9999	>0.9999
CuSO4 + M-PS 41.8 °C vs. CuSO4 + M-PS 49.4 °C	0.9260	0.9848	0.9967

Table S5: Exact adjusted P value resulted from Brown-Forsythe and Welch ANOVA test followed by post hoc Dunnett's T3 multiple comparison test of graphs A2-B2-C2 of Figure 5 (Analysis of swim bladder area).

Dunnett's T3 multiple comparisons test	Adjusted P Value A2	Adjusted P Value B2	Adjusted P Value C2
CTRL vs. $CuSO_4$	<0.0001	<0.0001	<0.0001
CTRL vs. $CuSO_4$ + M-PS 46.5 °C	<0.0001	0.0184	0.9850
CTRL vs. $CuSO_4$ + M-PS 49.9 °C	<0.0001	0.8912	0.0038
CTRL vs. $CuSO_4$ + M-PS 53.5 °C	<0.0001	0.3992	0.0264
$CuSO_4$ vs. $CuSO_4$ + M-PS 46.5 °C	0.0010	<0.0001	<0.0001
$CuSO_4$ vs. $CuSO_4$ + M-PS 49.9 °C	<0.0001	<0.0001	<0.0001
$CuSO_4$ vs. $CuSO_4$ + M-PS 53.5 °C	0.0005	<0.0001	<0.0001
$CuSO_4$ + M-PS 46.5 °C vs. $CuSO_4$ + M-PS 49.9 °C	0.5167	0.8272	0.2286
$CuSO_4$ + M-PS 46.5 °C vs. $CuSO_4$ + M-PS 53.5 °C	0.9762	0.9909	0.6942
$CuSO_4$ + M-PS 49.9 °C vs. $CuSO_4$ + M-PS 53.5 °C	0.9985	0.9999	0.9920

Table S6: Exact adjusted P value resulted from Brown-Forsythe and Welch ANOVA test followed by post hoc Dunnett's T3 multiple comparison test of graphs A1-B1 of Figure 6 (Analysis of operculum bone area).

Dunnett's T3 multiple comparisons test	Adjusted P Value A1	Adjusted P Value B1
CTRL vs. CuSO4	<0.0001	<0.0001
CTRL vs. CuSO4 + M-PS 36.6 °C	<0.0001	>0.9999
CTRL vs. CuSO4 + M-PS 41.8 °C	<0.0001	>0.9999
CTRL vs. CuSO4 + M-PS 49.4 °C	<0.0001	0.8922
CuSO4 vs. CuSO4 + M-PS 36.6 °C	<0.0001	<0.0001
CuSO4 vs. CuSO4 + M-PS 41.8 °C	<0.0001	<0.0001
CuSO4 vs. CuSO4 + M-PS 49.4 °C	<0.0001	<0.0001
CuSO4 + M-PS 36.6 °C vs. CuSO4 + M-PS 41.8 °C	0.9549	>0.9999
CuSO4 + M-PS 36.6 °C vs. CuSO4 + M-PS 49.4 °C	0.9998	0.9009
CuSO4 + M-PS 41.8 °C vs. CuSO4 + M-PS 49.4 °C	0.5660	0.9833

Table S7: Exact adjusted P value resulted from Brown-Forsythe and Welch ANOVA test followed by post hoc Dunnett's T3 multiple comparison test of graphs A2-B2 of Figure 6 (Analysis of operculum bone area).

Dunnett's T3 multiple comparisons test	Adjusted P Value A2	Adjusted P Value B2
CTRL vs. $CuSO_4$	<0.0001	<0.0001
CTRL vs. $CuSO_4$ + M-PS 46.5 °C	<0.0001	0.0558
CTRL vs. $CuSO_4$ + M-PS 49.9 °C	0.0004	0.1838
CTRL vs. $CuSO_4$ + M-PS 53.5 °C	0.0002	>0.9999
$CuSO_4$ vs. $CuSO_4$ + M-PS 46.5 °C	<0.0001	<0.0001
$CuSO_4$ vs. $CuSO_4$ + M-PS 49.9 °C	<0.0001	<0.0001
$CuSO_4$ vs. $CuSO_4$ + M-PS 53.5 °C	<0.0001	<0.0001
$CuSO_4$ + M-PS 46.5 °C vs. $CuSO_4$ + M-PS 49.9 °C	0.4781	>0.9999
$CuSO_4$ + M-PS 46.5 °C vs. $CuSO_4$ + M-PS 53.5 °C	0.3663	0.0886
$CuSO_4$ + M-PS 49.9 °C vs. $CuSO_4$ + M-PS 53.5 °C	>0.9999	0.2609

Table S8: Exact adjusted P value resulted from Brown-Forsythe and Welch ANOVA test followed by post hoc Dunnett's T3 multiple comparison test of graphs B1 of Figure 7 (Analysis of locomotor activity, 24 hours).

**Dunnett's T3 multiple comparisons test**	**Adjusted P Value B1**
CTRL vs. $CuSO_4$	<0.0001
CTRL vs. $CuSO_4$ + M-PS 36.6 °C	>0.9999
CTRL vs. $CuSO_4$ + M-PS 41.8 °C	0.9795
CTRL vs. $CuSO_4$ + M-PS 49.4 °C	0.6498
$CuSO_4$ vs. $CuSO_4$ + M-PS 36.6 °C	<0.0001
$CuSO_4$ vs. $CuSO_4$ + M-PS 41.8 °C	<0.0001
$CuSO_4$ vs. $CuSO_4$ + M-PS 49.4 °C	<0.0001
$CuSO_4$ + M-PS 36.6 °C vs. $CuSO_4$ + M-PS 41.8 °C	0.9992
$CuSO_4$ + M-PS 36.6 °C vs. $CuSO_4$ + M-PS 49.4 °C	0.9266
$CuSO_4$ + M-PS 41.8 °C vs. $CuSO_4$ + M-PS 49.4 °C	>0.9999

Table S9: Exact adjusted P value resulted from Brown-Forsythe and Welch ANOVA test followed by post hoc Dunnett's T3 multiple comparison test of graphs B2 of Figure 7 (Analysis of locomotor activity, 24 hours).

**Dunnett's T3 multiple comparisons test**	**Adjusted P Value B2**
CTRL vs. $CuSO_4$	<0.0001
CTRL vs. $CuSO_4$ + M-PS 46.5 °C	0.9847
CTRL vs. $CuSO_4$ + M-PS 49.9 °C	0.9868
CTRL vs. $CuSO_4$ + M-PS 53.5 °C	0.9942
$CuSO_4$ vs. $CuSO_4$ + M-PS 46.5 °C	<0.0001
$CuSO_4$ vs. $CuSO_4$ + M-PS 49.9 °C	<0.0001
$CuSO_4$ vs. $CuSO_4$ + M-PS 53.5 °C	<0.0001
$CuSO_4$ + M-PS 46.5 °C vs. $CuSO_4$ + M-PS 49.9 °C	>0.9999
$CuSO_4$ + M-PS 46.5 °C vs. $CuSO_4$ + M-PS 53.5 °C	>0.9999
$CuSO_4$ + M-PS 49.9 °C vs. $CuSO_4$ + M-PS 53.5 °C	>0.9999

Table S10: Exact adjusted P value resulted from Brown-Forsythe and Welch ANOVA test followed by post hoc Dunnett's T3 multiple comparison test of graphs B1 of Figure 8 (Analysis of locomotor activity, 48 hours).

**Dunnett's T3 multiple comparisons test**	**Adjusted P Value B1**
CTRL vs. $CuSO_4$	<0.0001
CTRL vs. $CuSO_4$ + M-PS 36.6 °C	0.9998
CTRL vs. $CuSO_4$ + M-PS 41.8 °C	0.9987
CTRL vs. $CuSO_4$ + M-PS 49.4 °C	0.1923
$CuSO_4$ vs. $CuSO_4$ + M-PS 36.6 °C	<0.0001
$CuSO_4$ vs. $CuSO_4$ + M-PS 41.8 °C	<0.0001
$CuSO_4$ vs. $CuSO_4$ + M-PS 49.4 °C	<0.0001
$CuSO_4$ + M-PS 36.6 °C vs. $CuSO_4$ + M-PS 41.8 °C	>0.9999
$CuSO_4$ + M-PS 36.6 °C vs. $CuSO_4$ + M-PS 49.4 °C	0.8054
$CuSO_4$ + M-PS 41.8 °C vs. $CuSO_4$ + M-PS 49.4 °C	0.7526

Table S11: Exact adjusted P value resulted from Brown-Forsythe and Welch ANOVA test followed by post hoc Dunnett's T3 multiple comparison test of graphs B2 of Figure 8 (Analysis of locomotor activity, 48 hours).

**Dunnett's T3 multiple comparisons test**	**Adjusted P Value B2**
CTRL vs. $CuSO_4$	<0.0001
CTRL vs. $CuSO_4$ + M-PS 46.5 °C	0.0124
CTRL vs. $CuSO_4$ + M-PS 49.9 °C	0.1503
CTRL vs. $CuSO_4$ + M-PS 53.5 °C	0.6910
$CuSO_4$ vs. $CuSO_4$ + M-PS 46.5 °C	<0.0001
$CuSO_4$ vs. $CuSO_4$ + M-PS 49.9 °C	<0.0001
$CuSO_4$ vs. $CuSO_4$ + M-PS 53.5 °C	<0.0001
$CuSO_4$ + M-PS 46.5 °C vs. $CuSO_4$ + M-PS 49.9 °C	0.9864
$CuSO_4$ + M-PS 46.5 °C vs. $CuSO_4$ + M-PS 53.5 °C	0.5308
$CuSO_4$ + M-PS 49.9 °C vs. $CuSO_4$ + M-PS 53.5 °C	0.9907

Table S12: Primer pairs used for RT-qPCR.

Gene name	GeneBank accession No	Primer sequences (5′ - 3′)
gapdh	NM_001115114	*Fw*: GTGGAGTCTACTGGTGTCTTC<br>*Rv:* GTGCAGGAGGCATTGCTTAC
mmp9	NM_213123	*Fw:* CATTAAAGATGCCCTGATGTATCCC<br>*Rv:* AGTGGTGGTCCGTGGTTGAG
sgk1	NM_199212	*Fw*: GGTAGCTTCGGCAAGGTTCT<br>*Rv:* CAGTAACACGTTGCGCTCTGA
socs3a	DQ333315.1	*Fw*: GGAAGACAAGAGCCGAGACT<br>*Rv:* GCGATACACACCAAACCCTG
